# Effects of Carbenoxolone on the Canine Pituitary-Adrenal Axis

**DOI:** 10.1371/journal.pone.0135516

**Published:** 2015-08-11

**Authors:** Takahiro Teshima, Hirotaka Matsumoto, Tomoko Okusa, Yumi Nakamura, Hidekazu Koyama

**Affiliations:** Division of Therapeutic Science I, Department of Veterinary Clinical Medicine, School of Veterinary Medicine, Faculty of Veterinary Science, Nippon Veterinary and Life Science University, 1-7-1 Kyonan-cho, Musashino-shi, Tokyo 180-8602, Japan; Uppsala University, SWEDEN

## Abstract

Cushing’s disease caused by pituitary corticotroph adenoma is a common endocrine disease in dogs. A characteristic biochemical feature of corticotroph adenomas is their relative resistance to suppressive negative feedback by glucocorticoids. The abnormal expression of 11beta-hydroxysteroid dehydrogenase (11HSD), which is a cortisol metabolic enzyme, is found in human and murine corticotroph adenomas. Our recent studies demonstrated that canine corticotroph adenomas also have abnormal expression of 11HSD. 11HSD has two isoforms in dogs, 11HSD type1 (HSD11B1), which converts cortisone into active cortisol, and 11HSD type2 (HSD11B2), which converts cortisol into inactive cortisone. It has been suggested that glucocorticoid resistance in corticotroph tumors is related to the overexpression of HSD11B2. Therefore it was our aim to investigate the effects of carbenoxolone (CBX), an 11HSD inhibitor, on the healthy dog’s pituitary-adrenal axis. Dogs were administered 50 mg/kg of CBX twice each day for 15 days. During CBX administration, no adverse effects were observed in any dogs. The plasma adrenocorticotropic hormone (ACTH), and serum cortisol and cortisone concentrations were significantly lower at day 7 and 15 following corticotropin releasing hormone stimulation. After completion of CBX administration, the HSD11B1 mRNA expression was higher, and HSD11B2 mRNA expression was significantly lower in the pituitaries. Moreover, proopiomelanocortin mRNA expression was lower, and the ratio of ACTH-positive cells in the anterior pituitary was also significantly lower after CBX treatment. In adrenal glands treated with CBX, HSD11B1 and HSD11B2 mRNA expression were both lower compared to normal canine adrenal glands. The results of this study suggested that CBX inhibits ACTH secretion from pituitary due to altered 11HSD expressions, and is potentially useful for the treatment of canine Cushing’s disease.

## Introduction

Corticotroph adenoma is the most common cause of canine Cushing’s disease, and the treatment options for dogs with Cushing’s disease are pituitary resection by hypophysectomy, radiotherapy, and medical management [1–3]. In humans, the typical treatment of Cushing’s disease is surgical resection of the pituitary tumor [4,5]. However, in veterinary medicine, the most common treatment is medical management, whereby trilostane and mitotane are most often used for the treatment [6,7]. These drugs can decrease circulating cortisol levels by inhibiting steroid synthesis (trilostane) or inducting adrenal gland necrosis (mitotane). The utilization and efficacy of these drugs has been well documented [8–12]. However, there are no reports that trilostane nor mitotane has curative effects on corticotroph adenoma. Moreover, use of these drugs may lead to the development of Nelson’s syndrome, as a consequence of suppressing cortisol negative feedback [13,14]. Our previous study found that pituitary size gradually enlarged and circulating adrenocorticotropic hormone concentrations increased via inhibited cortisol secretion after trilostane treatment in healthy dogs [15].

Recently, new drugs such as pasireotide and cabergoline, which are targeted at decreasing ACTH secretion from corticotroph tumors, have been studied for possible use in the management of human Cushing’s disease [16–19]. However, there is little research to support the direct targeting of canine corticotroph adenoma [20–22]. The candidates for the therapeutic agent of canine Cushing’s disease such as retinoic acid, pasireotide, and gefitinib, which are also targeted at decreasing ACTH secretion from corticotroph tumors. Retinoic acid and pasireotide have been reported that decreasing circulating ACTH concentrations and tumors size using dogs with Cushing’s disease [20,21].

Glucocorticoid resistance, which is a characteristic of corticotroph tumors, is partially caused by abnormal expression of 11-beta hydroxysteroid dehydrogenase (11HSD) [23,24]. 11HSD has two isoforms in humans, 11HSD type 1 (HSD11B1), which catalyzes the conversion of cortisone into active cortisol, and 11HSD type 2 (HSD11B2), which catalyzes the conversion of cortisol into inactive cortisone. Expression of both HSD11B1 and HSD11B2 have been documented in healthy dogs [25], and abnormal HSD11B1 and HSD11B2 expression patterns were found in canine corticotroph adenomas [26]. These findings are similar to those found in human and murine corticotroph adenomas [23,24,27]. A previous study using murine corticotroph tumor cells found that carbenoxolone (CBX), an 11HSD inhibitor, improved the negative feedback effect of glucocorticoids and enhanced apoptosis under existing cortisol levels [24]. However, the effect of CBX in dogs has not been studied. We aimed to investigate the effect of CBX on the pituitary–adrenal axis in healthy dogs.

## Materials and Methods

### Animals

Thirteen healthy Beagles (ORIENTAL YEAST, Tokyo, Japan) were randomly assigned to a control group or carbenoxolone administration group (CBX group). All dogs were male aged 1 to 3 years old (mean: 2.0 years old), and 8.9 to 12.4 kg body weight (mean: 10.2 kg). The control group contained six dogs, with a mean age of 2.3 years and a mean weight of 10.1 kg. The CBX group had seven dogs, with a mean age of 1.9 years and a mean weight of 10.5 kg. All dogs were individually housed at the same laboratory animal unit in separate pens (1.1x0.9 m) in temperature-controlled rooms with a 12-h light: 12-h dark cycle, with be provided small blanket and free access to water and fed a commercial dry food twice a day (PROSTAGE Pork & Rice, Yeaster, Hyogo, Japan, 25.9% crude protein, 12.6% fat, 48.1% carbohydrates, 4.0% fiber). All dogs were restricted to access outdoors, but could be outside of individual pens and interact with dogs when animal care staffs clean up dogs’ pens twice daily during experimental period. Protocols for all experiments involving the use of dogs were approved by the Bioethics Committee at Nippon Veterinary and Life Science University.

### CBX administration

In the CBX group, all dogs were administrated carbenoxolone disodium (LKT Laboratories, Inc., Saint Paul, MN, USA) capsuled orally twice a day with food. The dose given was 100mg/kg/day CBX for 15 days. During the administration period, general conditions were monitored daily.

### Blood sample collection and CRH-stimulation test

Blood samples were collected from the jugular vein and transferred to ice-chilled tubes containing EDTA and plain tubes. Plasma and serum were separated by centrifugation at 3000 rpm for 15 min at 4°C and stored at −80°C until assayed.

The CRH-stimulation test was performed by collecting blood samples for measurement of the ACTH concentration 0 and 15 min after intravenous administration of 1.5-μg ovine corticotropin-releasing factor (Peptide Institute, Inc., Osaka, Japan) per kg body weight. Samples were also collected for measurement of cortisol and cortisone concentration 0 and 30 min after administration of corticotropin-releasing factor [26].

The CRH-stimulation test was performed before, after 7 days, and following completion of CBX treatment. All CRH-stimulation tests were performed 4 h after the administration of the morning dose of CBX.

### Hormone determination

Plasma ACTH concentrations were measured by a solid-phase, 2-site chemiluminescent enzyme immunometric assay (ImmuliteACTH; Diagnostic Products Corporation, Los Angeles, CA, USA), as described previously [28]. The intra-assay coefficients of variation (CV) were 9.6% and 4.9% at ACTH levels of 5.3 and 221.8 pmol/l, respectively. The inter-assay CV were 8.8% and 5.1% at ACTH levels of 5.8 and 248.9 pmol/l, respectively.

Serum cortisol concentrations were measured by a competitive immunoassay (ImmuliteCortisol; Diagnostic Products Corporation), as described previously [29]. The intra-assay CV were 8.8% and 5.8% at cortisol levels of 74 and 524 nmol/l, respectively. The inter-assay CV were 10.0% and 6.3% at cortisol levels of 74 and 524 nmol/l, respectively.

Serum cortisone concentrations were measured by a chemiluminescent immunoassay (DetectX Cortisone; Arbor Assays LLC, Ann Arbor, MI, USA) in duplicate, as described previously [30]. The intra-assay CV were 5.7 and 7.9% at cortisone levels of 1.6 and 21.3 nmol/l, respectively. The inter-assay CV were 11.1 and 10.0% at cortisone levels of 1.8 and 21.3 nmol/l, respectively.

### Tissue samples

Each dog was euthanized by an intravenous overdose of pentobarbital solution following the final CRH-stimulation test. The pituitary and bilateral adrenal glands were carefully harvested and weighed before fixing in 4% paraformaldehyde for histological examination, or storage at −80°C for RNA extraction. Each pituitary was cut at the median sagittal plane and each adrenal gland was cut twice transversely, midway between the indentation of the phrenicoabdominal vein and each pole.

### Histological examination

Samples for histological examination were dehydrated, embedded in paraffin, and sections were cut at 4 μm. One section was stained with hematoxylin and eosin, and adjacent sections were subjected to immunohistochemical staining by standard enzyme antibody techniques using a monoclonal mouse antibody to synthetic ACTH1-39 (Dako Japan, Kyoto, Japan) [31].

### Pituitary morphometry

Pituitary morphometry was performed with Adobe Photoshop CS4 (Adobe Systems, San Jose, CA, USA). For each dog, the area and numbers of ACTH-positive cells in the anterior pituitary gland were measured. The anterior pituitary gland was divided equally into five regions between the dorsal and ventral ends of the pituitary gland in the median section, and two fields each were randomly selected from the five regions. The number of cells and the numbers of ACTH-positive cells per field of the anterior pituitary gland were measured in ten fields at 400× magnification for each dog. The ratio of ACTH-positive cells was calculated.

### RNA extraction and reverse transcription

Total RNA was extracted from pituitaries and adrenal glands in tubes containing 2-ml TRIzol (Invitrogen Japan, Tokyo, Japan). The tissues were disrupted using a standard homogenizer. For purification of total RNA, samples were treated with RNeasy Mini Kit (QIAGEN, Tokyo, Japan). The RNA yield was quantified by measuring the optical density of a sample diluted to 1:20 at 260 and 280 nm. One μg of total RNA of pituitary and adrenal gland were then reverse transcribed with the GoScript Reverse Transcriptase (Promega KK, Tokyo, Japan). Reverse transcribed samples were diluted to 20 μl in DEPC treated water.

### Real-time PCR

Real-time PCR analysis for HSD11B1, HSD11B2, and proopiomelanocortin (POMC), mRNAs were performed using the Applied Biosystems 7500 Sequence Detections System (Applied Biosystems Japan, Tokyo, Japan). Each primer sequence is shown in Table 1 [22,32]. The PCR reaction was performed with an end-volume of 20 μl. A master mix containing 0.5-μl reverse transcribed RNA, 10-μl 2× Go Taq qPCR Master Mix, 0.4 μl (4 pmol/l) of forward and reverse primers, and 8.7-μl DEPC-treated water per reaction was prepared. The PCR reaction was divided into two stages: (1) 2 min at 95°C, and (2) 3 s at 95°C, and 30 s at 60°C. Stage 2 was repeated 40 times. All samples were assayed in duplicate. The specificity and the size of the PCR products were assessed by adding a melt curve at the end of the amplifications and a single melting curve peak was observed. The relative quantitative analysis of each mRNA expression was performed using the delta-delta Ct method using three genes (GAPDH, GUS, and RPS5) as an internal control.

**Table 1:** Primer sequences using in this study.

Gene	Sequence (5’→3’)	
HSD11B1	For	AAGGTCAATGTGTCGATCACTCTCT
	Rev	ATCCCAGAAACGGCCTTCAT
HSD11B2	For	GGGTCAAGGTCAGCGTCATC
	Rev	CTCCCAATGTCCCACATTCC
POMC	For	GGCCTCTGTGGAAGTGAGTG
	Rev	ACGCCAGCAGGTTACTTTCC
GAPDH	For	GATGGGCGTGAACCATGAG
	Rev	TCATGAGGCCCTCCACGAT
GUS	For	CCTCCTGCCGTATTACCCTTG
	Rev	TCTGGACGAAGTAACCCTTGG
RPS5	For	TCACTGGTGAGAACCCCCT
	Rev	CCTGATTCACACGGCGTAG

### Statistical analysis

All results are presented as mean ± SD. Differences between control and CBX groups before CBX administration were determined by two-sided Mann-Whitney’s *U* test. The changes in plasma ACTH concentration, serum cortisol and cortisone concentrations, and cortisol-to-cortisone levels were analyzed with two-way ANOVA followed by Tukey-Kramer’s post hoc tests. The ratio and area of ACTH-positive cells in the anterior pituitary gland and the expression of mRNA were compared by two-tailed Mann–Whitney *U* test. Statistical analyses were performed using Excel 2010 (Microsoft, Redmond, WA, USA) with add-in software Statcel 3. Differences were considered significant when *P* < 0.05.

## Results

### General condition

During CBX administration, adverse effects such as lethargy, anorexia, vomiting, and diarrhea were not observed in any dogs. Results of complete blood counts and serum biochemical profiles were not different during CBX treatment (Appendix 1).

### ACTH, cortisol, cortisone concentrations, and cortisol-to-cortisone ratios

ACTH, cortisol, and cortisone concentrations and cortisol-to-cortisone ratios of both basal and CRH-stimulated were not significantly different between control and the CBX groups at day 0 (Table 2).

**Table 2:** Comparison of ACTH, cortisol, and cortisone concentrations, and cortisol-to-cortisone ratio before CBX treatment.

	Control	CBX
ACTH (pmol/l)		
Basal concentration	4.8±1.8	4.3±1.4
CRH-stimulated concentration	27.9±2.5	28.8±2.3
Cortisol (nmol/l)		
Basal concentration	128±35	131±39
CRH-stimulated concentration	275±30	269±36
Cortisone (nmol/l)		
Basal concentration	29.1±5.4	28.5±4.9
CRH-stimulated concentration	39.3±3.1	38.8±2.0
Cortisol-to-cortisone ratio		
Basal concentration	4.4±0.7	4.6±0.7
CRH-stimulated concentration	7.2±1.1	7.0±1.0

**Fig 1:**
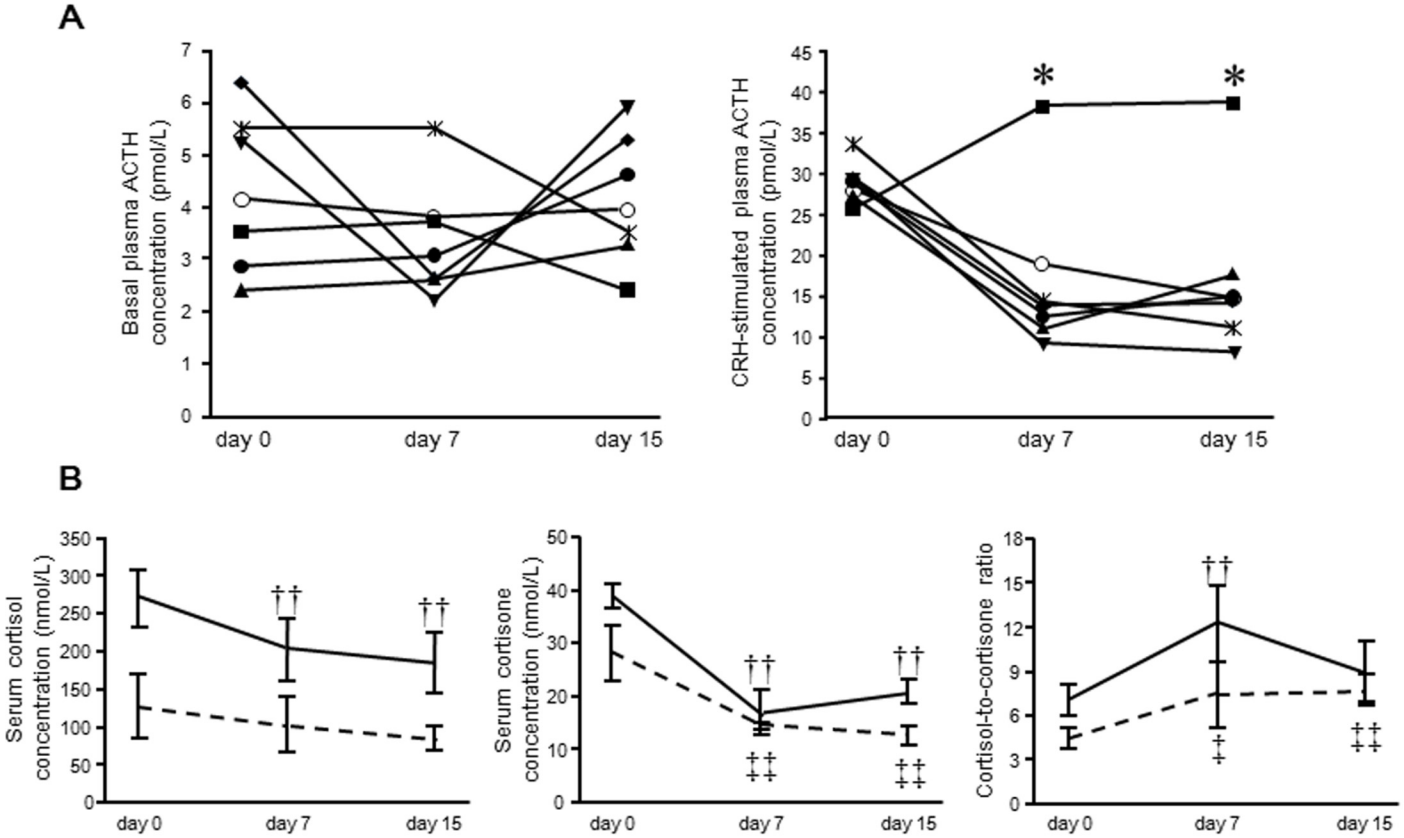
Effects of a 100mg/kg bodyweight dose of CBX on plasma ACTH, serum cortisol and cortisone concentrations, and cortisol-to-cortisone ratio at basal and after CRH- stimulation. (A) Each line represents one dog treated with carbenoxolone. Basal-plasma-ACTH concentrations are not significantly changed during carbenoxolone administration. One dog had increased CRH-stimulated-plasma-ACTH concentrations. The CRH-stimulated-plasma-ACTH concentrations at day 7 and 15 are significantly decreased compared with those of day 0. Error bar is S.D. * *P* < 0.05 *vs* day 0. (B) The CRH-stimulated serum cortisol concentrations are gradually **decreased**. Basal and CRH-stimulated serum cortisone concentrations are decreased significantly compared with those of day 0. The cortisol-to cortisone ratios during carbenoxolone administration are increased compared with day 0. The dashed line indicates the basal concentrations, and the solid line indicates the CRH-stimulated concentrations. Error bars are S.D. †† *P* < 0.01 *vs* day 0 of basal concentration, ‡*P* < 0.05 *vs* day 0 of CRH-stimulated concentration, and ‡‡ *P* < 0.01 *vs* day 0 of CRH-stimulated concentration.

Basal plasma ACTH concentrations were not significantly altered throughout CBX administration (Fig 1A). Following CRH stimulation, plasma ACTH concentrations at day 7 and 15 were significantly lower than those on day 0 (Fig 1A) (*P* < 0.05). Serum CRH-stimulated cortisol and cortisone concentrations were significantly lower than those on day 0 (*P* < 0.01) (Fig 1B). The cortisol-to-cortisone ratios during CBX administration were significantly higher than those on day 0 (Fig 1B). One dog had gradually increasing CRH-stimulated ACTH concentrations, but cortisol and cortisone levels during CBX administration were lower than those on day 0.

### Pituitary HSD11B1, HSD11B2, and POMC mRNA expression

**Fig 2:**
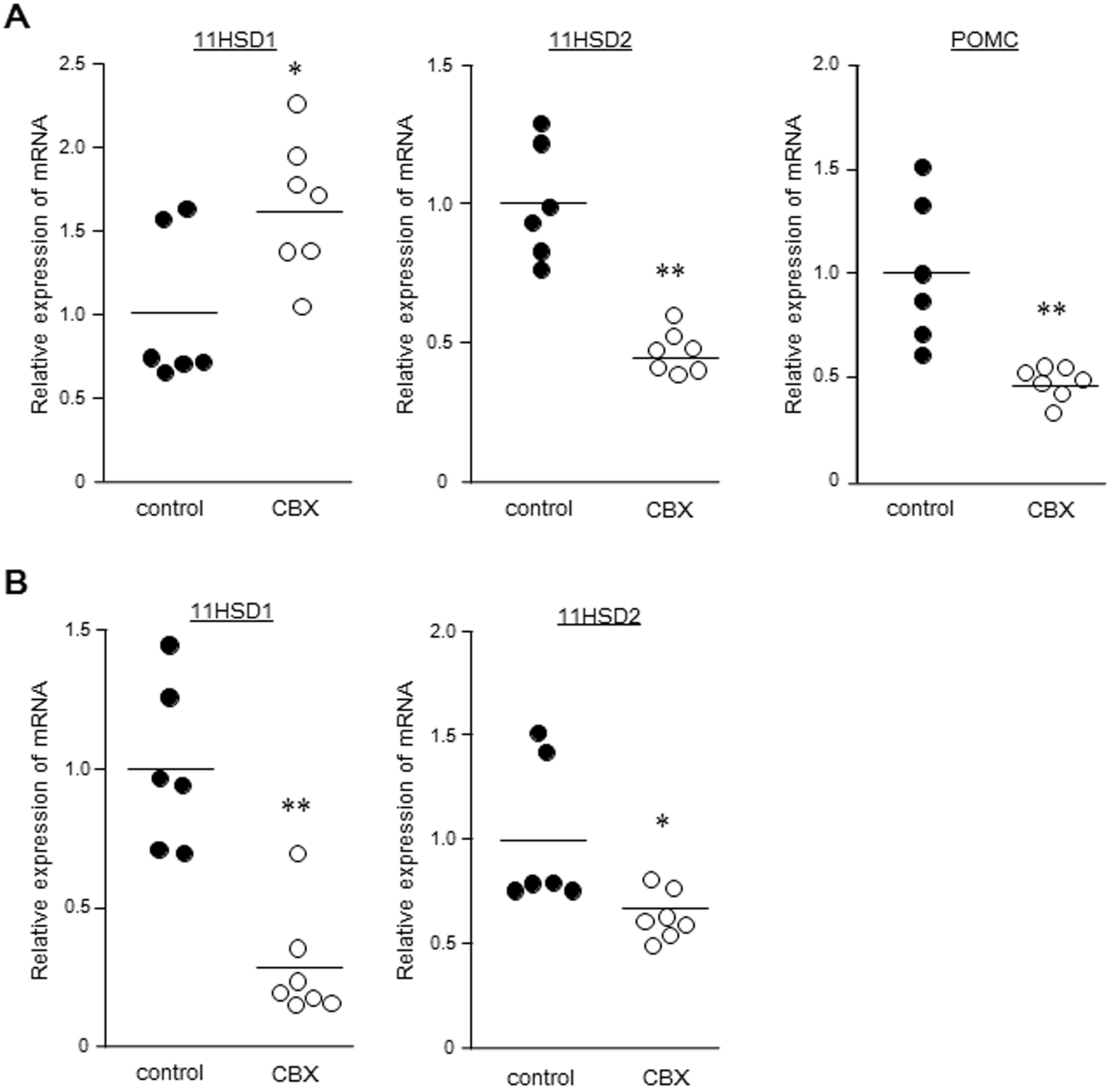
The relative HSD11B1, HSD11B2, and POMC mRNA expressions after CBX treatment. (A) HSD11B1 mRNA expression in pituitary glands treated with carbenoxolone is greater than the control group, but HSD11B2 and POMC mRNA expressions are lower significantly. (B) The relative HSD11B1 and HSD11B2 mRNA expressions in the adrenal gland. HSD11B1 and HSD11B2 mRNA expressions of the CBX group are significantly lower than the control group. The solid line indicates the average, and each circle represents a dog. * *P* < 0.05 *vs* control group. ** *P* < 0.01 *vs* control group.

After 15 days of treatment with 100-mg/kg/day CBX, the relative HSD11B1 mRNA expression in the CBX group (1.66 ± 0.35) was greater than that of control (*P* < 0.05) (Fig 2A). In contrast, the HSD11B2 mRNA level in the CBX group (0.47 ± 0.07) was significantly lower than that of the control group (*P* < 0.01). The POMC mRNA level in the CBX group (0.47 ± 0.07) was lower than that of the control group (*P* < 0.01).

### Adrenal HSD11B1 and HSD11B2 mRNA expression

The HSD11B1 mRNA expression in the CBX group (0.28 ± 0.18) was significantly lower than that of the control group (*P* < 0.01) (Fig 2B). The HSD11B2 mRNA expression in the CBX group (0.63 ± 0.10) was also significantly lower that of the control group (*P* < 0.05) (Fig 2B).

### ACTH-positive cells in the anterior pituitary

**Fig 3:**
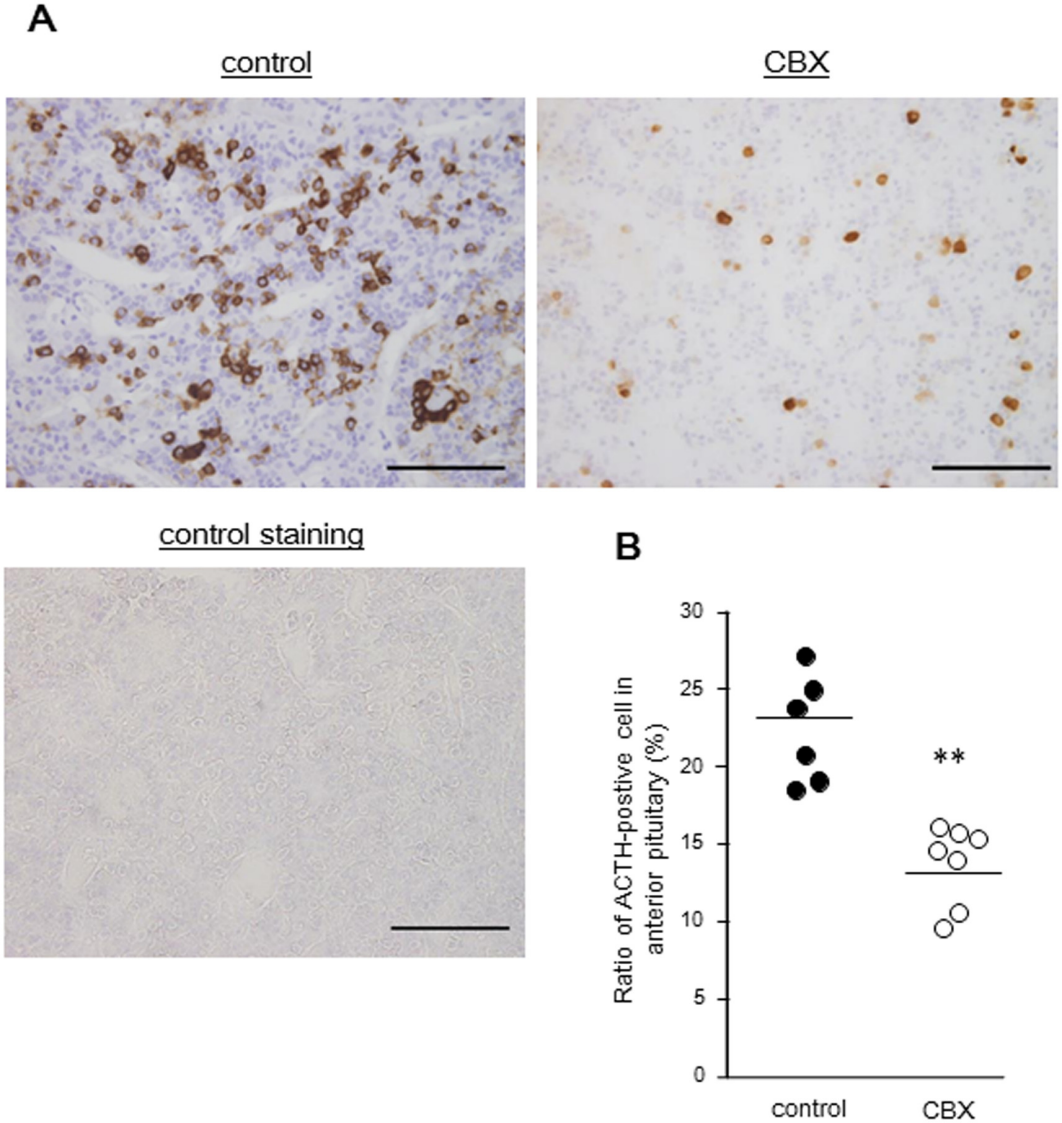
Changes of ACTH-positive cells in the anterior pituitary after CBX treatment. (A) Immunohistochemistry for ACTH are performed in anterior pituitary of control and treated with CBX dogs. Control staining is performed by the same method without a monoclonal mouse antibody to synthetic ACTH1-39 (Dako Japan, Kyoto, Japan). Bars = 100 μm. (B) The comparison of ratio of ACTH-positive cell in anterior pituitary between control and treated with carbenoxolone. The ratio of ACTH-positive cells is significantly lower in treated with carbenoxolone. The solid line indicates the average, and each circle represents a dog. ** *P* < 0.01 vs control group.

After CBX administration, the ratio of ACTH-positive cells in the CBX group (13.7 ± 2.4%) was significantly lower than that of the control group (22.3 ± 3.2%) (*P* < 0.01) (Fig 3A and 3B). There was no significant change in ACTH-positive cells area between the CBX and control groups.

## Discussion

The effects of CBX on dogs have not previously been reported, aside from one study that examined its effects on canine gastric mucosal permeability and blood flow [32]. As a consequence, there is no information on the effects of CBX on canine pituitary–adrenal axis or blood biochemistry. In humans, there are some reports that prolonged CBX treatment for gastritis and peptic ulcer can lead to pseudohyperaldosteronism [33]. In our study, neither hypokalemia, hypernatremia, nor other apparent changes in blood chemistry were not observed in dogs treated with CBX. During CBX administration, side effects such as lethargy, anorexia, vomiting, and diarrhea were also not observed.

CBX, a synthetic analogue of glycyrrhetinic acid, is a potent 11HSD inhibitor, and it has been reported that CBX is a more specific and potent HSD11B2 inhibitor than HSD11B1 in human and rat [34]. However, some reports found that inhibiting HSD11B1 via CBX causes glucose homeostasis problems and obesity [35–37]. Other studies have investigated the effects of CBX on HSD11B2 [24,33]. In human study, it was reported that treatment for 7 days with 300 mg/day of CBX resulted in progressively lower plasma cortisol levels over the course of treatment [34]. A study using db/db mice found that CBX dose-dependently decreased HSD11B1 activity [35]. In humans, orally administered CBX was rapidly absorbed from the gut and 80% was present in the plasma 2 h later. The half-life of CBX was found to be about 19 h [38]. However, there is no information about the oral administration nor metabolism of CBX in dogs. Therefore, we decided to administer 100mg/kg/day CBX using as a reference the dosage of CBX in a previous murine study [35]. In dogs, secretion of ACTH is episodic and pulsatile in nature, following a diurnal rhythm in healthy dogs and those with Cushing’s disease [39,40]. In addition, many types of stress can stimulate ACTH secretion. Therefore, we examined secretory function of ACTH in the pituitary by determining plasma ACTH concentrations after CRH-stimulation.

After initiation of CBX treatment, basal-plasma-ACTH concentrations were not significantly different, but CRH-stimulated-ACTH concentrations were significantly decreased. One of the seven dogs had gradually increasing CRH-stimulated ACTH levels, but cortisol and cortisone decreased in line with other dogs. It is unclear why one dog had higher ACTH secretion, but we hypothesize that the sensitivity of 11-HSD to CBX to is different in this dog compared with others. In the CBX group, the ratio of cortisol-to-cortisone increased during treatment. However, overall serum cortisol and cortisone concentrations decreased during CBX treatment. These results suggest that CBX more potently inhibited HSD11B2 activity, because cortisone decreased more than cortisol.

After completion of treatment with CBX, HSD11B1 mRNA expression was greater than that of the control group, whereas HSD11B2 mRNA expression was lower than that of the control group. These results are contrary to our previous results on the pattern of 11HSD mRNA expression in canine corticotroph adenoma (HSD11B1 mRNA expression was lower and HSD11B2 mRNA expression was higher than that of normal canine corticotroph cells) [26]. POMC mRNA expression in the CBX group was significantly lower than that of the control group. This change is similar to the results of murine corticotroph tumor cells treated with CBX [24]. Therefore, CBX had an inhibitory effect on ACTH secretion and POMC expression in the presence of cortisol. Moreover, we found that the ratio of ACTH-positive cells in the anterior pituitary significantly decreased after administration of CBX. In adrenal glands treated with CBX, both of HSD11B1 and HSD11B2 mRNA expressions were lower than that of normal adrenal glands. Therefore the pattern of HSD11B1 in adrenal glands treated with CBX was different from that in pituitaries. It cannot be determined from our study why the HSD11B1 mRNA expression in pituitary was increased, but it might be that the decreased feedback of cortisol was responsible, or the sensitivity of CBX to HSD11B1 might be different among tissues. To clarify these postulations, further studies are needed.

In conclusion, previously observed side effects, including pseudohyperaldosteronism, and changes in blood chemistry were not observed during CBX administration in healthy dogs. Orally administration of CBX induced not only decreases in ACTH and cortisol secretion, but also ACTH-positive cell ratios in the anterior pituitary. These changes are probably caused by, in part, the inhibition of HSD11B2. Glucocorticoid resistance in corticotroph tumors, which is the characteristic of Cushing’s disease, partially results from intracellular inactivation of cortisol to cortisone by overexpression of HSD11B2 [23,24]. Therefore, our findings suggest that CBX may have a beneficial effect of decreasing HSD11B2 expression and ACTH secretion in canine pituitary.

## Supporting Information

S1 Table Changes in blood tests and blood chemistry during carbenoxolone treatment. All parameters are not changed significantly during CBX administration.
(PDF)
