# Supplementary material for: Effects of Carbenoxolone on the Canine Pituitary-Adrenal Axis
Source: PLoS One. 2015 Aug 11;10(8):e0135516. doi: 10.1371/journal.pone.0135516 (PMC4532459; doi:10.1371/journal.pone.0135516)
Supplement: S1 Table — All parameters are not changed significantly during CBX administration. (PDF) [file pone.0135516.s001.pdf]

	reference range	Day 0	Day 7	Day 15
RBC (x10^3/μ l)	550–850	716±26	730±27	716±38
Hb (g/dl)	12.0–18.0	15.4±0.5	15.9±0.6	15.9±1.0
Hct (%)	37.0–55.0	45.4±1.9	46.5±1.5	44.0±4.0
MCV (fl)	60–77	63±2	64±1	61±4
MCH (pg)	19.5–26.0	21.6±0.1	21.9±0.2	22.2±0.5
MCHC (g/dl)	32.0–36.0	34.0±0.9	34.2±0.5	36.2±2.8
WBC (x10^3/μ l)	5.7–16.3	10.3±1.1	11.5±2.3	10.7±2.3
PLT (x10^3/μ l)	164–510	351±28	370±40	369±37
TP (g/dl)	5.0–7.2	6.3±0.1	6.3±0.3	6.1±0.3
Alb (g/dl)	2.6–4.0	3.3±0.1	3.2±0.1	3.1±0.2
AST (U/l)	17–44	29±6	32±5	32±3
ALT (U/l)	17–78	49±12	56±17	52±17
ALP (U/l)	47–254	170±54	189±63	195±66
GGT (U/l)	5–14	9±1	9±1	9±2
T-Bil (mg/dl)	0.1–0.5	0.3±0.07	0.3±0.06	0.3±0.07
BUN (mg/dl)	9.2–29.2	12.6±2.8	15.7±3.5	13.8±1.5
Cre (mg/dl)	0.4–1.4	0.5±0.11	0.6±0.07	0.5±0.07
Glu (mg/dl)	75–128	105±6	102±7	102±7
T-Cho (mg/dl)	111–312	128±7	133±9	134±20
TG (mg/dl)	30–133	26±8	30±9	25±6
Ca (mg/dl)	9.3–12.1	10.4±0.7	10.7±0.8	10.6±0.6
Na (mEq/l)	141–152	145±2	145±2	144±1
K (mEq/l)	3.8–5.0	4.1±0.3	4.2±0.2	4.4±0.1
Cl (mEq/l)	102–117	115±1	112±3	115±2
